# Preconception telomere length as a novel maternal biomarker to assess the risk of spina bifida in the offspring

**DOI:** 10.1002/bdr2.1682

**Published:** 2020-05-02

**Authors:** Damiat Aoulad Fares, Sarah Schalekamp‐Timmermans, Tim S. Nawrot, Régine P. M. Steegers‐Theunissen

**Affiliations:** ^1^ Department of Obstetrics and Gynaecology Erasmus University Medical Center Rotterdam The Netherlands; ^2^ Department of Environmental Sciences Hasselt University Hasselt Belgium; ^3^ Department of Public Health & Primary Care Leuven University Leuven Belgium

**Keywords:** aging, folic acid, lifestyle, neural tube defects, nutrition, oxidative stress, telomere length

## Abstract

**Background:**

Periconception interactions between maternal conditions and environmental and genetic factors are involved in the pathogenesis and prevention of neural tube defects (NTD), such as spina bifida. These factors have in common that they can impair the oxidative pathway, resulting in excessive (chronic) oxidative stress and inflammation.

**Methods:**

Review of the literature concerning underlying mechanisms and biomarkers of aging particularly during reproduction. A number of molecular markers for biological aging have been identified, including telomere length (TL). Excessive telomere shortening is an index of senescence, causes genomic instability and is associated with a higher risk of age‐related diseases. Furthermore, TL shortening is associated with the similar environmental and lifestyle exposures associated with NTD risk.

**Results:**

Embryonic mice deficient in the telomerase gene show shorter TL and failure of closure of the neural tube as the main defect, suggesting that this developmental process is among the most sensitive to telomere loss and chromosomal instability.

**Conclusions:**

From this background, we hypothesize that preconceptional long term exposure to harmful environmental and lifestyle risk factors accelerates a woman's aging process, which can be measured by TL, and thereby her underlying risk of NTD offspring. Alternatively, it might be that women with an increased NTD risk already exhibit a more advanced biological age before the onset of pregnancy compared to women of identical calendar age.

## 1 INTRODUCTION

Neural tube defects (NTDs) are severe birth defects involving the central nervous system. They arise from incomplete closure of the neural tube during the first weeks of embryogenesis. Worldwide birth prevalence is approximately one in 1,000 births (Mitchell et al., 2004).

NTDs have an enormous impact not only on the affected child, parents and family, but also on society because of high societal and health care costs for life long medical treatment and support.

One of the most common type of NTDs in humans is spina bifida. Spina bifida is a complex disease caused by a combination of genetic and periconception maternal environmental factors that can induce excessive oxidative stress and inflammation (Groenen et al., 2003). Maternal obesity, inositol deficiency, poor nutrition and lifestyle, hyperhomocysteinemia, and the use of antifolates are modifiable environmental factors that are involved in the pathogenesis of spina bifida (Carmichael, Rasmussen, & Shaw, 2010; Groenen et al., 2003; Mitchell et al., 2004; Steegers‐Theunissen, Boers, Trijbels, & Eskes, 1991; Vajda & Eadie, 2005; Vujkovic et al., 2009). Maternal folate status has been proven to be of great importance in the pathogenesis of NTDs. Folate, an anti‐oxidant in natural form, is an important substrate of the one carbon metabolism and thereby essential for processes such as lipid, protein, DNA synthesis and repair, but also for DNA methylation. Prior environmental factors have in common that they can impair the oxidative pathway, resulting in excessive oxidative stress.

Embryogenesis in very early pregnancy is sensitive to excessive oxidative stressors of which a mild to moderate increased plasma homocysteine concentration is a sensitive marker (Steegers‐Theunissen, Twigt, Pestinger, & Sinclair, 2013), including the development and folding of the neural tube. Therefore, the identification of a stable marker of the preconception oxidative stress status in women is of major importance in the prediction and prevention of the future risk of NTD in the offspring.

There is an increasing interest in aging during reproduction (Herrmann, Pusceddu, Marz, & Herrmann, 2018). A number of molecular markers for biological aging have been identified, including telomere length (TL). Telomeres are nucleoprotein structures that cap the end of chromosomes and thereby protect it from degradation. Excessive telomere shortening is an index of senescence, causes genomic instability and is associated with a higher risk of age‐related diseases, like cardiovascular disease and type 2 diabetes mellitus. Furthermore, TL shortening is associated with exposure to environmental and lifestyle factors that can induce oxidative stress and inflammation (Sahin et al., 2011). Embryonic mice deficient in the telomerase gene show shorter TL and failure of closure of the neural tube as the main defect, suggesting that this developmental process is among the most sensitive to telomere loss and chromosomal instability (Herrera, Samper, & Blasco, 1999).

**FIGURE 1:**
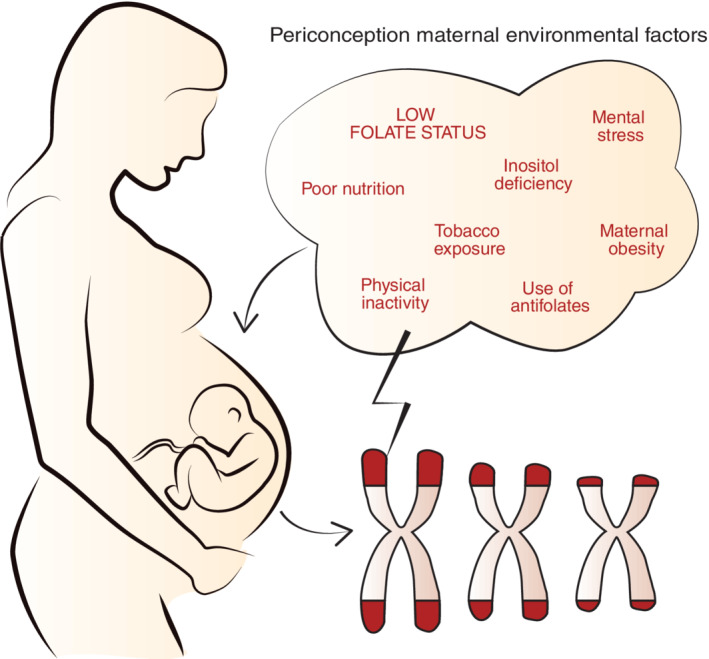
Hypothesis: preconception chronic exposure to environmental and lifestyle risk factors accelerates a woman's aging process resulting in telomere length shortening, which is associated with an increased risk of spina bifida in the offspring

We hypothesize that preconception TL shortening in woman, due to chronic excessive exposure to oxidative stressors, such as poor nutrition and lifestyle, is associated with an increased risk of spina bifida in the offspring. Regarding to this hypothesis, we reviewed the current evidence (Figure 1).

## 2 METHODS

### 2.1 Aging

Aging is a complex physiological process reactive to health conditions, environmental factors, behavior, and genetic background. Even though biological aging is universal and unavoidable the process does not occur in a uniform way. Known the complexity of the biological aging process, there is no single and simple measure of an individual's aging process.

The aging process can be split in to two distinct types of aging: chronological aging and biological aging. In which, the aging process of chronological aging is defined by age calculated in years and occurs at a constant rate for each individual. Biological age describes the functional status of the body relative to its chronological age and occurs at a different rate for each individual. The rate of aging is an interplay between underlying mechanisms involving damaging processes and the action of defense and repair mechanisms (Martens, 2018).

Multiple markers for prediction of biological age and the rate of aging have been reported. Blood pressure, fasting glucose, glycated hemoglobin (HbA1C), intima media thickness, and number of nephrons appear to be among those. Blood biomarkers are increasingly used to predict an individual's biological age independent of its calendar age (Herrmann et al., 2018).

One of the key aspects of aging is genomic instability. Low folate status and a mild to moderate hyperhomocysteinemia can impair cell multiplication, DNA synthesis and programming due to changes of the epigenome, which can result in genomic instability (Steegers‐Theunissen et al., 2013). Other hallmarks of aging that causes damage to cellular function include, inter alia, epigenetic alterations, deregulated nutrient sensing, mitochondrial dysfunction, and attrition of telomeres. Telomeres represent the protective end caps of chromosomes that are of critical importance for genomic integrity and stability. Over the course of each cell division, TL shortens. TL has been proposed as a biomarker for biological age, its association with age is confirmed in large population‐based studies (Martens, 2018).

### 2.2 Telomeres and telomerase

Human telomeres span several kilobase (kb) tandem repeated TTAGG sequences with a 3′G‐rich single stranded overhang. Telomeres prevent unwanted recombination and degradation of chromosomal ends. In addition, loss of coding DNA is prevented during DNA replication (Herrmann et al., 2018).

In humans, TL shortens in somatic cells with age due to the increased amounts of cellular divisions. TL is maintained by the cellular ribonucleoprotein enzyme telomerase. Telomerase adds telomeric repeat sequences to the end of chromosomes and is mostly active in germ, stem and immortal cells, and mainly repressed in somatic cells (Blackburn, Epel, & Lin, 2015).

DNA binding proteins are able to bind with telomeres to form the shelterin‐complex. The proteins of the shelterin‐complex are involved in the control of telomere length by regulating the access of telomerase to the G‐strand overhang and by protecting it from degradation. In addition, end‐to‐end fusions of chromosomes are prevented (Blackburn et al., 2015; Martens, 2018). Telomerase inhibition is influenced by the amount of shelterin complexes on telomeres (Martens, 2018).

Most of the large population‐based studies is focused on leukocyte TLs. It has been shown that leukocyte TL is highly correlated with TL of other somatic tissues from the same individual such as muscle, fat, skin, and synovial tissue. This indicates that a clear intra‐individual synchronization in TL exists in adults (Daniali et al., 2013).

### 2.3 Oxidative stress, inflammation, and telomere length

The intricacy of TL translates in a high inter‐individual variability, when comparing same‐aged people (Muezzinler, Zaineddin, & Brenner, 2013). Both external and internal factors can interact with telomeres and may influence TL through life. Predominantly, external and internal factors that increase the oxidative stress or inflammatory status of an individual have been associated with shortening of TL. Von Zglinicki, Saretzki, Docke, and Lotze (1995) were the first to show experimentally in 1995, that cultivating human fibroblasts under hypoxia conditions (represented as a state of oxidative stress) indeed shortened telomeres. Another study showed that the G‐rich parts of the telomere sequence (TTAGGG) in human fibroblasts are highly sensitive for DNA damage induced by oxidative stress conditions (Kawanishi & Oikawa, 2004). Additionally, an experimental study showed that mice models of chronic inflammation induces telomere dysfunction due to increased oxidative stress (Jurk et al., 2014).

TL shortening has been associated with tobacco smoke exposure (Valdes et al., 2005), obesity (Valdes et al., 2005), life stress (Epel et al., 2004), physical inactivity (Arsenis, You, Ogawa, Tinsley, & Zuo, 2017), and exposure to air pollution (Pieters et al., 2016). Recent findings showed that newborn TL sets adult TL (Bijnens et al., 2017) and shorter TL (Martens et al., 2017) in newborns is associated with prenatal pregnancy body mass index (BMI) (Martens, Plusquin, Gyselaers, De Vivo, & Nawrot, 2016), prenatal exposure to air pollution and folic acid status (Louis‐Jacques et al., 2016). Paul et al. (2015) showed that folate status influences TL by affecting DNA integrity through DNA methylation.

Mechanisms by which TL may be influenced by these factors are mostly explained by the direct or indirect effects of these factors on the oxidative and inflammatory status of humans.

Similarly, cellular aging is affected by mental stress through oxidative stress and telomerase activity. Highly stressed women are characterized by lower telomerase activity and higher oxidative stress compared to women with a low stress level (Epel et al., 2004). In addition, regular physical activity has been associated with decreased levels of oxidative stress and inflammation (Epel et al., 2004).

This gives emphasis to the vulnerability of telomeres for oxidative stress and inflammation, as described previously.

### 2.4 Telomere length and age‐related diseases

Short telomeres and telomere dysfunction, independently of age have been linked to numerous age‐related diseases. All these diseases are characterized by an accelerated rate of telomere shortening.

Large population based studies identify that subjects with shorter telomeres were characterized by a significantly higher hazard ratio for all‐cause mortality compared to those with longer TL (Mons et al., 2017).

There is evidence that reduced TL is associated with elevated risk for future age‐related disease, including: cardiovascular disease (Haycock et al., 2014), atherosclerosis (Fitzpatrick et al., 2007), myocardial infarction (Fitzpatrick et al., 2007), type 2 diabetes mellitus (Willeit et al., 2014), and Alzheimer's disease (Zhan et al., 2015). To conclude large population‐based study results propose that TL potentially may be predictive of lifespan and longevity independent of age (Martens, 2018). Upon the observational findings, experimental evidence revealed that late‐generation telomerase knock‐out mice (Terc‐KO) with critically short telomeres exhibited an aging phenotype associated with p53 activation, suppression of master regulators of mitochondrial biology, ventricular dilation, myocardial thinning, cardiac dysfunction, and sudden death (Sahin et al., 2011). Therefore, TL might not just be a marker of the aging process but might play a fundamental biological role within the core axis of aging.

## 3 DISCUSSION

TL shortening is associated with variations in folate status, exposure to environmental and lifestyle factors that can induce oxidative stress and inflammation. Of great interest is that these conditions in women are also associated with a significantly increased risk of having a child with spina bifida. These environmental factors have in common that they can generate excessive amounts of reactive oxidative radicals resulting in excessive chronic oxidative stress. Interestingly, a large meta‐analysis found a high and very consistent heritability estimate for TL, with stronger effects from maternal to offspring (Broer et al., 2013). Thereby, embryogenesis in very early pregnancy is very sensitive to excessive oxidative stress, including the development and folding of the neural tube.

Herrera et al. (1999) showed that mice deficient in the telomerase gene show defects in the closure of the neural tube. The frequency of NTD in mouse deficient in the telomerase gene suggests a role for TL and telomere loss from chromosome ends. Cells, derived from embryos that lack mouse telomerase RNA and that are telomerase‐deficient, of mice that fail to close the neural tube have significantly shorter TL than mice of the same embryos but with a closed neural tube. Furthermore, an increased apoptosis and decreased viability was shown in cells derived from NTD affected embryos. This association between a decreased TL and NTD strongly suggests that the neural tube closure defect may be a consequence of telomere shortening to a critical length.

During embryonic development telomerase is highly active, directly after birth it is down‐regulated. Remarkably, the highest levels of human telomerase RNA in human embryos are detected at the central nervous system, specifically in the primitive neurepithelial cells of the neural tube.

The main defect detected in these embryos is the closure of the neural tube, suggesting that the neural tube formation is among the processes most sensitive to TL shortening during development. Perchance due to the massive proliferation that occurs during early development for the formation of the central nervous system. Foregoing implies an important role for TL during the neural tube formation and explains the occurrence of the phenotype (Herrera et al., 1999).

Several nutritional factors like vitamins, minerals, and other bioactive dietary components are able to directly or indirectly influence TL through several mechanisms. Recent studies have shown consistent associations between TL and the availability of B and D vitamins, serum folate, and its metabolites. Anti‐oxidant activity, DNA methylation, and prevention of DNA damage are the most important mechanisms through which these nutritional factors slow down telomere attrition. In summary, a healthy lifestyle with a diet rich in fruits and vegetables combined with exercise, lower BMI and no smoking is associated with longer telomeres (Arsenis et al., 2017; Epel et al., 2004; Valdes et al., 2005) In this line, whereas high homocysteine levels increases oxidative stress, an association was found between high levels of homocysteine and shortening of telomeres in the presence of systemic inflammation (Pusceddu et al., in press; Shin & Baik, 2016).

The hypothetic role of telomeres length in NTDs pathogenesis is illustrated by the example of the epidemiological and biological evidence of the association between mild to moderate maternal hyperhomocysteinemia and the increased risk of spina bifida offspring (Groenen et al., 2003; Steegers‐Theunissen et al., 1991).

Plasma homocysteine is an intermediate of 1‐C metabolism and a sensitive biomarker of oxidative stress. Mild to moderate hyperhomocysteinemia is associated with impairment of biological processes involved in cell proliferation, programming, and apoptosis. Hyperhomocysteinemia induces global and gene specific hypomethylation, impairs the synthesis of proteins, lipids and DNA, reduces DNA repair, and increases the production of reactive oxidative species (Steegers‐Theunissen et al., 2013). From this evidence we hypothesize that mild to moderate hyperhomocysteinemia is involved in the pathophysiology of NTD by reducing the synthesis or increasing the damage of the DNA of the telomeres and or by impairment of the programming due to global or gene specific hypomethylation of telomerase. This hypothesis is supported by Cecchini et al. (2019) reporting that homocysteine in the developing spinal cord causes changes in cell proliferation, adhesion, induces apoptosis and that it alters arrangement of the spinal cord layers. Li et al. (2019) showed that homocysteine induces changes in gene and protein expression of astrocytes of the neural tissue. In addition, intracellular folate deficiency underlying mild to moderate hyperhomocysteinemia, shortens TL and damages telomeric DNA. (Bull et al., 2014; Li et al., 2019).

Finally, other associations between TL and obstetric outcomes have been reported. Telomerase activity is decreased or absent in placentas of fetal growth restricted newborns (Fragkiadaki et al., 2016). Similarly, TL shortening has been reported in combination with an increased formation of telomere aggregates in trophoblastic cells from pregnancies complicated by preeclampsia (Sukenik‐Halevy et al., 2016).

Evidence of advanced maternal age and NTD occurrence in offspring is limited. A meta‐analysis, however, showed an increased risk of having an offspring with NTDs for mothers 40 years of age or older, with the strongest effect for spina bifida (Vieira & Castillo Taucher, 2005). These findings are similar with other studies that show a higher NTD in offspring prevalence among mothers in older age groups (Au, Ashley‐Koch, & Northrup, 2010; Eggink & Steegers‐Theunissen, 2020; Li et al., 2006; Sipek et al., 2002; Zheng et al., 2007). Notable is that there is also evidence for mothers between 14 and 20 years old having a higher risk for a child with spina bifida. An explanation for this could be the fact that most age‐associated shortening occurs during rapid somatic expansions, as occurs from birth through puberty (Sidorov, Kimura, Yashin, & Aviv, 2009). Together with earlier discussed neural tube formation being among the most sensitive processes to TL shortening during development.

We hypothesize that preconceptional maternal exposure to environmental risk factors accelerates the aging process, which can be measured by TL, and thereby her underlying risk of NTD offspring. Alternatively, it might be that women with an increased NTD risk already exhibit a more advanced biological age before the onset of pregnancy compared to women of identical calendar age.

Investigating TL in the woman as a marker of chronic oxidative stress, induced by variation in folate supply, poor nutrition, obesity, and other environmental exposures, could serve as a novel preconception biomarker in the future. By this means the risk of spina bifida offspring may be assessed and modified by a more personalized preconception treatment, for example, folic acid supplement use, dietary pattern, lifestyle, and so forth.

## CONFLICT OF INTEREST

The authors report no conflict of interest.

## AUTHOR CONTRIBUTIONS

R. S. T. initiated the hypothesis and D. A. reviewed the review data and wrote the first version of the article. S. S., R. S. T., and T. N. contributed to the design of the paper, cowriting of the article, revisions, and gave input at all stages of the study. All authors have approved the final version of the manuscript.

## Data Availability

In this manuscript no ‘Expects Data’ or ‘Mandates Data’ is used, therefore the data availability statement is not applicable.

## References

[bdr21682-bib-0001] Arsenis, N. C. , You, T. , Ogawa, E. F. , Tinsley, G. M. , & Zuo, L. (2017). Physical activity and telomere length: Impact of aging and potential mechanisms of action. Oncotarget, 8(27), 45008–45019. 28410238 10.18632/oncotarget.16726 PMC5546536

[bdr21682-bib-0002] Au, K. S. , Ashley‐Koch, A. , & Northrup, H. (2010). Epidemiologic and genetic aspects of spina bifida and other neural tube defects. Developmental Disabilities Research Reviews, 16(1), 6–15. 20419766 10.1002/ddrr.93 PMC3053142

[bdr21682-bib-0003] Bijnens, E. M. , Zeegers, M. P. , Derom, C. , Martens, D. S. , Gielen, M. , Hageman, G. J. , … Nawrot, T. S. (2017). Telomere tracking from birth to adulthood and residential traffic exposure. BMC Medicine, 15(1), 205. 29157235 10.1186/s12916-017-0964-8 PMC5697215

[bdr21682-bib-0004] Blackburn, E. H. , Epel, E. S. , & Lin, J. (2015). Human telomere biology: A contributory and interactive factor in aging, disease risks, and protection. Science, 350(6265), 1193–1198. 26785477 10.1126/science.aab3389

[bdr21682-bib-0005] Broer, L. , Codd, V. , Nyholt, D. R. , Deelen, J. , Mangino, M. , Willemsen, G. , … Boomsma, D. I. (2013). Meta‐analysis of telomere length in 19,713 subjects reveals high heritability, stronger maternal inheritance and a paternal age effect. European Journal of Human Genetics, 21(10), 1163–1168. 23321625 10.1038/ejhg.2012.303 PMC3778341

[bdr21682-bib-0006] Bull, C. F. , Mayrhofer, G. , O'Callaghan, N. J. , Au, A. Y. , Pickett, H. A. , Low, G. K. , … Fenech, M. F. (2014). Folate deficiency induces dysfunctional long and short telomeres; both states are associated with hypomethylation and DNA damage in human WIL2‐NS cells. Cancer Prevention Research (Philadelphia, PA), 7(1), 128–138. 10.1158/1940-6207.CAPR-13-0264 24253316

[bdr21682-bib-0007] Carmichael, S. L. , Rasmussen, S. A. , & Shaw, G. M. (2010). Prepregnancy obesity: A complex risk factor for selected birth defects. Birth Defects Research. Part A, Clinical and Molecular Teratology, 88(10), 804–810. 20973050 10.1002/bdra.20679

[bdr21682-bib-0008] Cecchini, M. S. , Bourckhardt, G. F. , Jaramillo, M. L. , Ammar, D. , Muller, Y. M. R. , & Nazari, E. M. (2019). Exposure to homocysteine leads to cell cycle damage and reactive gliosis in the developing brain. Reproductive Toxicology, 87, 60–69. 31082465 10.1016/j.reprotox.2019.05.054

[bdr21682-bib-0009] Daniali, L. , Benetos, A. , Susser, E. , Kark, J. D. , Labat, C. , Kimura, M. , … Aviv, A. (2013). Telomeres shorten at equivalent rates in somatic tissues of adults. Nature Communications, 4, 1597. 10.1038/ncomms2602 PMC3615479 23511462

[bdr21682-bib-0010] Eggink, A. J. , & Steegers‐Theunissen, R. P. M. (2020). Neural tube anomalies: An update on the pathophysiology and prevention In Johnson A., Oepkes D., & Kilby M. D. (Eds.), Fetal therapy: Scientific basis and critical appraisal of clinical benefits (2nd ed., pp. 449–455). Cambridge, UK: Cambridge University Press.

[bdr21682-bib-0011] Epel, E. S. , Blackburn, E. H. , Lin, J. , Dhabhar, F. S. , Adler, N. E. , Morrow, J. D. , & Cawthon, R. M. (2004). Accelerated telomere shortening in response to life stress. Proceedings of the National Academy of Sciences of the United States of America, 101(49), 17312–17315. 15574496 10.1073/pnas.0407162101 PMC534658

[bdr21682-bib-0012] Fitzpatrick, A. L. , Kronmal, R. A. , Gardner, J. P. , Psaty, B. M. , Jenny, N. S. , Tracy, R. P. , … Aviv, A. (2007). Leukocyte telomere length and cardiovascular disease in the cardiovascular health study. American Journal of Epidemiology, 165(1), 14–21. 17043079 10.1093/aje/kwj346

[bdr21682-bib-0013] Fragkiadaki, P. , Tsoukalas, D. , Fragkiadoulaki, I. , Psycharakis, C. , Nikitovic, D. , Spandidos, D. A. , & Tsatsakis, A. M. (2016). Telomerase activity in pregnancy complications (review). Molecular Medicine Reports, 14(1), 16–21. 27175856 10.3892/mmr.2016.5231 PMC4918539

[bdr21682-bib-0014] Groenen, P. M. , Peer, P. G. , Wevers, R. A. , Swinkels, D. W. , Franke, B. , Mariman, E. C. , & Steegers‐Theunissen, R. P. (2003). Maternal myo‐inositol, glucose, and zinc status is associated with the risk of offspring with spina bifida. American Journal of Obstetrics and Gynecology, 189(6), 1713–1719. 14710103 10.1016/s0002-9378(03)00807-x

[bdr21682-bib-0015] Haycock, P. C. , Heydon, E. E. , Kaptoge, S. , Butterworth, A. S. , Thompson, A. , & Willeit, P. (2014). Leucocyte telomere length and risk of cardiovascular disease: Systematic review and meta‐analysis. BMJ, 349, g4227. 25006006 10.1136/bmj.g4227 PMC4086028

[bdr21682-bib-0016] Herrera, E. , Samper, E. , & Blasco, M. A. (1999). Telomere shortening in mTR−/− embryos is associated with failure to close the neural tube. The EMBO Journal, 18(5), 1172–1181. 10064584 10.1093/emboj/18.5.1172 PMC1171208

[bdr21682-bib-0017] Herrmann, M. , Pusceddu, I. , Marz, W. , & Herrmann, W. (2018). Telomere biology and age‐related diseases. Clinical Chemistry and Laboratory Medicine, 56(8), 1210–1222. 29494336 10.1515/cclm-2017-0870

[bdr21682-bib-0018] Jurk, D. , Wilson, C. , Passos, J. F. , Oakley, F. , Correia‐Melo, C. , Greaves, L. , … von Zglinicki, T. (2014). Chronic inflammation induces telomere dysfunction and accelerates ageing in mice. Nature Communications, 2, 4172. 10.1038/ncomms5172 PMC4090717 24960204

[bdr21682-bib-0019] Kawanishi, S. , & Oikawa, S. (2004). Mechanism of telomere shortening by oxidative stress. Annals of the New York Academy of Sciences, 1019, 278–284. 15247029 10.1196/annals.1297.047

[bdr21682-bib-0020] Li, W. , Ma, Y. , Li, Z. , Lv, X. , Wang, X. , Zhou, D. , … Huang, G. (2019). Folic acid decreases astrocyte apoptosis by preventing oxidative stress‐induced telomere attrition. International Journal of Molecular Sciences, 21(1), 62. 10.3390/ijms21010062 PMC6981374 31861819

[bdr21682-bib-0021] Li, Z. , Ren, A. , Zhang, L. , Ye, R. , Li, S. , Zheng, J. , … Li, Z. (2006). Extremely high prevalence of neural tube defects in a 4‐county area in Shanxi Province, China. Birth Defects Research. Part A, Clinical and Molecular Teratology, 76(4), 237–240. 16575897 10.1002/bdra.20248

[bdr21682-bib-0022] Louis‐Jacques, A. F. , Salihu, H. M. , King, L. M. , Paothong, A. , Sinkey, R. G. , Pradhan, A. , … Whiteman, V. E. (2016). A positive association between umbilical cord RBC folate and fetal TL at birth supports a potential for fetal reprogramming. Nutrition Research, 36(7), 703–709. 27269132 10.1016/j.nutres.2016.01.009

[bdr21682-bib-0023] Martens, D. (2018). Telomere biology in early life and its environmental determinants. Hasselt, Belgium: University of Hasselt.

[bdr21682-bib-0024] Martens, D. S. , Cox, B. , Janssen, B. G. , Clemente, D. B. P. , Gasparrini, A. , Vanpoucke, C. , … Nawrot, T. S. (2017). Prenatal air pollution and newborns' predisposition to accelerated biological aging. JAMA Pediatrics, 171(12), 1160–1167. 29049509 10.1001/jamapediatrics.2017.3024 PMC6233867

[bdr21682-bib-0025] Martens, D. S. , Plusquin, M. , Gyselaers, W. , De Vivo, I. , & Nawrot, T. S. (2016). Maternal pre‐pregnancy body mass index and newborn telomere length. BMC Medicine, 14(1), 148. 27751173 10.1186/s12916-016-0689-0 PMC5067896

[bdr21682-bib-0026] Mitchell, L. E. , Adzick, N. S. , Melchionne, J. , Pasquariello, P. S. , Sutton, L. N. , & Whitehead, A. S. (2004). Spina bifida. Lancet, 364(9448), 1885–1895. 10.1016/S0140-6736(04)17445-X 15555669

[bdr21682-bib-0027] Mons, U. , Muezzinler, A. , Schottker, B. , Dieffenbach, A. K. , Butterbach, K. , Schick, M. , … Brenner, H. (2017). Leukocyte telomere length and all‐cause, cardiovascular disease, and cancer mortality: Results from individual‐participant‐data meta‐analysis of 2 large prospective cohort studies. American Journal of Epidemiology, 185(12), 1317–1326. 28459963 10.1093/aje/kww210 PMC5860628

[bdr21682-bib-0028] Muezzinler, A. , Zaineddin, A. K. , & Brenner, H. (2013). A systematic review of leukocyte telomere length and age in adults. Ageing Research Reviews, 12(2), 509–519. 23333817 10.1016/j.arr.2013.01.003

[bdr21682-bib-0029] Paul, L. , Jacques, P. F. , Aviv, A. , Vasan, R. S. , D'Agostino, R. B. , Levy, D. , & Selhub, J. (2015). High plasma folate is negatively associated with leukocyte telomere length in Framingham offspring cohort. European Journal of Nutrition, 54(2), 235–241. 24793435 10.1007/s00394-014-0704-1 PMC4218881

[bdr21682-bib-0030] Pieters, N. , Janssen, B. G. , Dewitte, H. , Cox, B. , Cuypers, A. , Lefebvre, W. , … Nawrot, T. S. (2016). Biomolecular markers within the Core Axis of aging and particulate air pollution exposure in the elderly: A cross‐sectional study. Environmental Health Perspectives, 124(7), 943–950. 26672058 10.1289/ehp.1509728 PMC4937852

[bdr21682-bib-0031] Pusceddu, I. , Herrmann, W. , Kleber, M. E. , Scharnagl, H. , Hoffmann, M. M. , Winklhofer‐Roob, B. M. , März, W., & Herrmann, M. (in press). Subclinical inflammation, telomere shortening, homocysteine, vitamin B6, and mortality: The Ludwigshafen risk and cardiovascular health study. European Journal of Nutrition. 10.1007/s00394-019-01993-8 PMC7230054 31129702

[bdr21682-bib-0032] Sahin, E. , Colla, S. , Liesa, M. , Moslehi, J. , Muller, F. L. , Guo, M. , … DePinho, R. A. (2011). Telomere dysfunction induces metabolic and mitochondrial compromise. Nature, 470(7334), 359–365. 21307849 10.1038/nature09787 PMC3741661

[bdr21682-bib-0033] Shin, C. , & Baik, I. (2016). Leukocyte telomere length is associated with serum vitamin B12 and Homocysteine levels in older adults with the presence of systemic inflammation. Clinical Nutrition Research, 5(1), 7–14. 26839872 10.7762/cnr.2016.5.1.7 PMC4731864

[bdr21682-bib-0034] Sidorov, I. , Kimura, M. , Yashin, A. , & Aviv, A. (2009). Leukocyte telomere dynamics and human hematopoietic stem cell kinetics during somatic growth. Experimental Hematology, 37(4), 514–524. 19216021 10.1016/j.exphem.2008.11.009

[bdr21682-bib-0035] Sipek, A. , Horacek, J. , Gregor, V. , Rychtarikova, J. , Dzurova, D. , & Masatova, D. (2002). Neural tube defects in The Czech Republic during 1961‐1999: Incidences, prenatal diagnosis and prevalences according to maternal age. Journal of Obstetrics and Gynaecology, 22(5), 501–507. 12521417 10.1080/0144361021000003636

[bdr21682-bib-0036] Steegers‐Theunissen, R. P. , Boers, G. H. , Trijbels, F. J. , & Eskes, T. K. (1991). Neural‐tube defects and derangement of homocysteine metabolism. The New England Journal of Medicine, 324(3), 199–200. 1984202 10.1056/NEJM199101173240315

[bdr21682-bib-0037] Steegers‐Theunissen, R. P. , Twigt, J. , Pestinger, V. , & Sinclair, K. D. (2013). The periconceptional period, reproduction and long‐term health of offspring: The importance of one‐carbon metabolism. Human Reproduction Update, 19(6), 640–655. 23959022 10.1093/humupd/dmt041

[bdr21682-bib-0038] Sukenik‐Halevy, R. , Amiel, A. , Kidron, D. , Liberman, M. , Ganor‐Paz, Y. , & Biron‐Shental, T. (2016). Telomere homeostasis in trophoblasts and in cord blood cells from pregnancies complicated with preeclampsia. American Journal of Obstetrics and Gynecology, 214(2), e281–e283. 10.1016/j.ajog.2015.08.050 26321036

[bdr21682-bib-0039] Vajda, F. J. , & Eadie, M. J. (2005). Maternal valproate dosage and foetal malformations. Acta Neurologica Scandinavica, 112(3), 137–143. 16097954 10.1111/j.1600-0404.2005.00458.x

[bdr21682-bib-0040] Valdes, A. M. , Andrew, T. , Gardner, J. P. , Kimura, M. , Oelsner, E. , Cherkas, L. F. , Oelsner, E. , Aviv, A. , & Spector, T. D. (2005). Obesity, cigarette smoking, and telomere length in women. Lancet, 366(9486), 662–664. 16112303 10.1016/S0140-6736(05)66630-5

[bdr21682-bib-0041] Vieira, A. R. , & Castillo Taucher, S. (2005). Edad materna y defectos del tubo neural: evidencia para un efecto mayor en espina bifida que anencefalia [Maternal age and neural tube defects: Evidence for a greater effect in spina bifida than in anencephaly]. Revista Medica de Chile, 133(1), 62–70. 15768151 10.4067/s0034-98872005000100008

[bdr21682-bib-0042] von Zglinicki, T. , Saretzki, G. , Docke, W. , & Lotze, C. (1995). Mild hyperoxia shortens telomeres and inhibits proliferation of fibroblasts: A model for senescence? Experimental Cell Research, 220(1), 186–193. 7664835 10.1006/excr.1995.1305

[bdr21682-bib-0043] Vujkovic, M. , Steegers, E. A. , Looman, C. W. , Ocke, M. C. , van der Spek, P. J. , & Steegers‐Theunissen, R. P. (2009). The maternal Mediterranean dietary pattern is associated with a reduced risk of spina bifida in the offspring. BJOG, 116(3), 408–415. 19187373 10.1111/j.1471-0528.2008.01963.x

[bdr21682-bib-0044] Willeit, P. , Raschenberger, J. , Heydon, E. E. , Tsimikas, S. , Haun, M. , Mayr, A. , … Kiechl, S. (2014). Leucocyte telomere length and risk of type 2 diabetes mellitus: New prospective cohort study and literature‐based meta‐analysis. PLoS One, 9(11), e112483. 25390655 10.1371/journal.pone.0112483 PMC4229188

[bdr21682-bib-0045] Zhan, Y. , Song, C. , Karlsson, R. , Tillander, A. , Reynolds, C. A. , Pedersen, N. L. , & Hagg, S. (2015). Telomere length shortening and Alzheimer disease–a Mendelian randomization study. JAMA Neurology, 72(10), 1202–1203. 26457630 10.1001/jamaneurol.2015.1513

[bdr21682-bib-0046] Zheng, X. Y. , Song, X. M. , Chen, G. , Chen, J. P. , Ji, Y. , Wu, J. L. , … Fan, X. H. (2007). Epidemiology of birth defects in high‐prevalence areas of China. Zhonghua Liu Xing Bing Xue Za Zhi, 28(1), 5–9. 17575922

